# Diagnosis and clinical presentation of iliac graft-enteric fistula: A case report

**DOI:** 10.1016/j.ijscr.2024.110460

**Published:** 2024-10-20

**Authors:** Fereshte Maghsoudlou, Reza Zahedpasha, Parisa Nikkhoo, Pezhman Kharazm, Mohammad Hadi Gharib, Maryam Shahali Ramsheh

**Affiliations:** a Department of Radiology, School of Medicine, 5th Azar Hospital, Gorgan, Golestan, Iran; b Golestan University of Medical Sciences, Gorgan, Golestan, Iran; c Clinical Research Development Center, 5th Azar Medical Center, Golestan University of Medical Sciences, Gorgan, Iran; d Vascular Surgery, Clinical Research Development Center, 5th Azar Medical Center, Golestan University of Medical Sciences, Gorgan, Iran

**Keywords:** Case-report, CT scan, Diagnosis, Digestive system fistula, Gastrointestinal hemorrhage, Iliac graft-enteric fistula

## Abstract

**Introduction:**

Aorto-enteric fistula (AEF) is a life-threatening complication arising from abnormal connections between the gastrointestinal tract and major arteries. One uncommon type, iliac artery-enteric fistula (IEF), can occur following vascular interventions such as arterial stent-graft placement.

**Case presentation:**

We report the case of a 47-year-old male presenting with hematemesis and abdominal pain, who was diagnosed with an iliac graft-enteric fistula. Timely recognition and management were crucial for a favorable outcome.

**Clinical discussion:**

Diagnosing AEFs remains challenging, requiring a multidisciplinary approach and high clinical suspicion. While computed tomography angiography (CTA) is commonly used for diagnosis, its sensitivity may be limited, emphasizing the importance of integrating clinical history and findings. Management strategies vary based on etiology and patient status, with surgery being pivotal.

**Conclusion:**

Aorto-enteric fistula, which can arise from a thrombosed graft, presents diagnostic challenges due to its rare formation. In patients with a history of vascular interventions and gastrointestinal bleeding, AEF should be considered. This case underscores the need for heightened awareness among healthcare professionals regarding AEF diagnosis and management to reduce severe morbidity, mortality, and prolonged hospital stays.

## Highlights

• Consider Aorto-enteric fistula in GI bleeding post-vascular surgery.
• Use CT angiography for accurate diagnosis confirmation.
• A thorough vascular surgery history aids in early diagnosis.
• Symptoms often include gastrointestinal bleeding, hematemesis, and melena.

## 1 Introduction

The term “Aorto-enteric fistula” (ArEF) refers to a broad category of fistulations that occur between the gastrointestinal tract (GI tract) and the major arteries. These include iliac artery-enteric fistula (IEF), aorto-esophageal, and aorto-gastric fistula [1]. It is an uncommon yet severe complication that might result in significant gastrointestinal bleeding and life-threatening risks [2].

Primary AEFs are less common than secondary AEFs and can be brought on by aneurysms, infections, cancer, or the ingestion of foreign objects [3]. Secondary AEFs frequently arise as a result of iatrogenic consequences, namely after the repair of abdominal aortic aneurysms (AAA) utilizing synthetic grafts, as well as following vascular interventions such as arterial bypass surgery, endovascular procedures, or arterial stent-graft [1,4,5].

Most AEFs occur at the proximal anastomotic site of the aorta in the abdomen and the third or fourth parts of the duodenum, with an occurrence rate of approximately 1 % or even lower following aortic surgery. IEFs are uncommon subtypes of AEFs [6].

AEFs are linked to a significant death rate (ranging from 65 % to 100 %) and morbidity. The death rate is 100 % in the absence of surgical intervention. Published evidence is scarce regarding diagnosing this uncommon yet potentially fatal illness [7].

Given the need to appropriately identify and treat this condition, we believe that increasing the number of reported cases with comparable manifestations will assist physicians in considering this diagnosis and avoiding overlooking any patients. Thus, this study reported a 47-year-old male patient who came with a suspicious history of hematemesis and abdominal pain and was diagnosed with an iliac graft-enteric fistula.

## 2 Case presentation

A 47-year-old male patient with a history of hypertension, Protein C deficiency, and lower limb ischemia. He underwent a bypass surgery from aorta to right common iliac- left common femoral artery using a bifurcated graft two years ago. The pre-operative CTA was indicative of complete para renal aorta thrombosis (including right renal artery). Run off was visible on right common iliac and left common femoral arteries. One month after the surgery, he underwent an endovascular intervention and stent graft placement in graft's right limb- iliac artery anastomoses because of a para-anastomotic pseudo aneurysm. 3 months later, he experienced an acute ischemia in the right lower limb following thrombosis of the right limb of the graft (according to CTA) and finally the right lower extremity was amputated from below the knee. 3 months later, he presented with generalized abdominal pain, pallor, suspicious history of hematemesis and melena. Initial evaluation revealed a blood pressure of 105/55 mmHg, heart rate of 115 beats per minute, oxygen saturation (SpO2) of 94 %, and a respiratory rate of 18 breaths per minute. Laboratory tests showed a red blood cell (RBC) count of 1.77× 106/ml, hemoglobin (HB) level of 3 g/dl, white blood cell (WBC) count of 9.1× 103/uL with 84 % neutrophils, and platelet (PLT) count of 596× 103/uL, Blood Urea Nitrogen (BUN) of 25 mg/dl, and Creatinine of 1.7 mg/dl.

After admission to the emergency department, the patient received 3 units of packed red blood cells (PRBC) and 2 units of fresh frozen plasma (FFP) due to a hemoglobin level of 3 g/dl. Subsequently, a computed tomography (CT) scan was performed, revealing characteristics indicative of the need for surgical intervention.

**Fig. 1:**
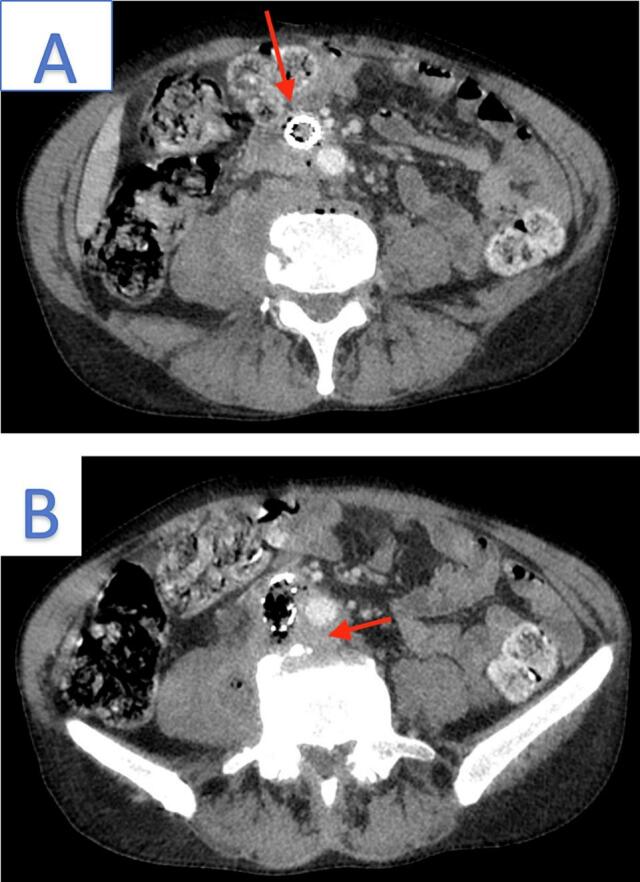
A: The arrow highlights iliac artery stent thrombosis, circumferential thickening, and ectopic gas. B: The arrow indicates abnormal soft tissue thickening with a poorly defined boundary between the iliac stent and prevertebral soft tissue.

**Fig. 2:**
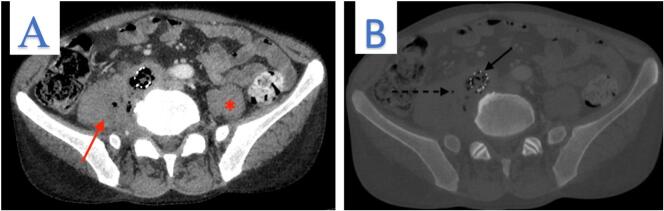
A: Arrow denotes heterogeneous enlargement of the right psoas muscle compared to the normal left psoas muscle. B: Arrow indicates a thrombotic iliac stent containing a gas bubble, while the dashed arrow highlights the presence of ectopic gas.

The CT scan with intravenous (IV) contrast revealed thrombosis of right iliac artery stent which contains air bubbles, along with circumferential wall thickening of graft and ectopic gas (Fig. 1A). In addition, there were poor plane of demarcation with adjacent vertebral body due to abnormal prevertebral soft tissue thickening, and inflammatory changes (Fig. 1B).The right psoas muscle was heterogeneously enlarged with a collection that was filled with fluid and air bubbles measuring 67 × 40 × 37 mm (Fig. 2).

**Fig. 3:**
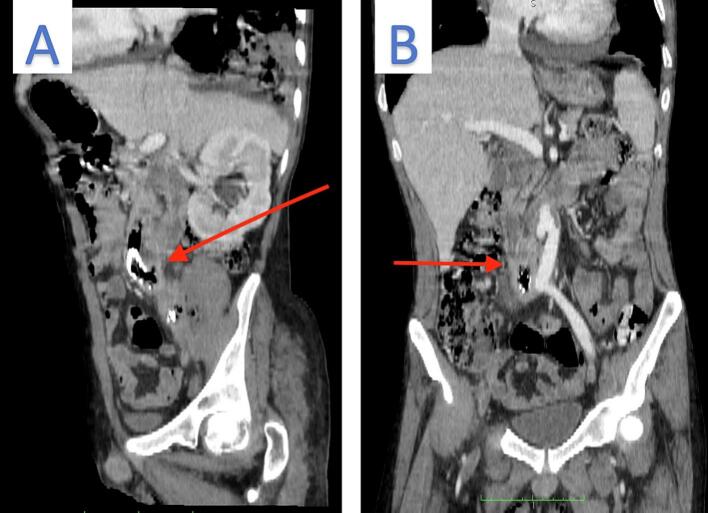
A: In sagittal view, the arrow indicates irregularity and thickening of this segment of the duodenum, with a suspicious connection noted on this side. A: In coronal view, the arrow identifies a fluid-filled structure as the third part of the duodenum, situated near the thrombosed stent.

**Fig. 4:**
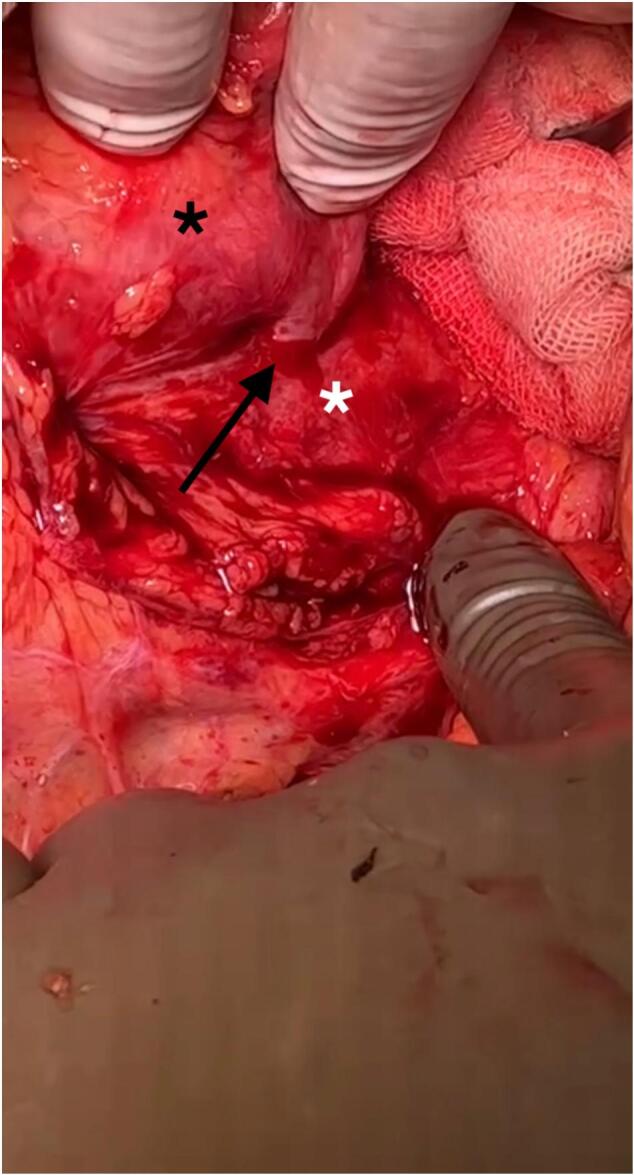
The black arrow indicates the Iliac-enteric fistula (IEF), the white star marks the artery, and the black star denotes the duodenum.

For a more detailed review images underwent reconstruction in coronal and sagittal planes, which demonstrated tubular fluid-filled structure in close vicinity to the right iliac artery graft, that was actually the 3rd part of duodenum. On closer inspection we found mild irregularity and thickening in this portion of duodenum associated with loss of fat plane interface with the mentioned thrombosed graft (Fig. 3A). according to these findings and clinical course of upper GI bleeding we suggested that, the iliac artery stent is complicated with a duodenal (D3/D4) fistula, leading to abscess formation extending to the right psoas muscle and prevertebral soft tissue (Fig. 3B). Our suspicion was conformed by surgery, and the final diagnosis was secondary iliac graft-enteric fistula (Fig. 4).

**Fig. 5:**
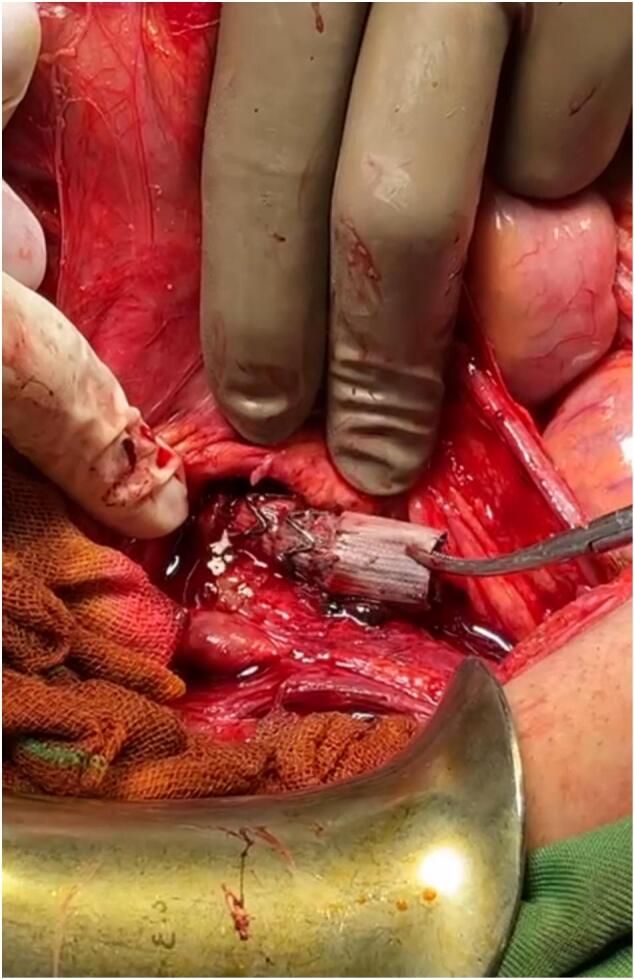
An iliac artery stent graft is visible.

In our case, following the thrombosis of the stent in the right iliac artery, a fistula developed, leading to the formation of an abscess and upper GI bleeding. The patient underwent resuscitation and was administered two FFP units and three packed RBC units. Additionally, antibiotics were prescribed, and the patient went through surgery to repair the fistula connecting the iliac and the duodenum (Fig. 5). The graft stent was extracted, and the thrombosed graft limb was surgically removed together with the infected tissues.

In short, the patient was discharged from the hospital in a stable state after receiving a total of 14 units of packed RBC and 10 units of FFP over a period of 14 days.

For the patient's follow-up, it was recommended to perform an upper gastrointestinal endoscopy and CT angiography of the aorta, iliac arteries, and left lower extremity six months post-discharge. Subsequently, these interventions are to be conducted annually.

No issues were detected during the six-month and eighteen-month follow-ups.

## 3 Discussion

Aortoenteric fistulas (AEFs) are rare but potentially fatal conditions characterized by an unusual connection between the aorta and the digestive system. The occurrence of this condition is notably infrequent, with studies suggesting it affects only a small fraction of patients [8]. This predominantly male-affecting disorder, typically seen in older individuals but with a wide age range, can be caused by various factors including aortic abnormalities, foreign bodies, tumors, radiation therapy, and specific infections [9,10]. Experts theorize that the formation of these fistulas involves physical pressure and inflammatory processes occurring between the affected aorta and nearby digestive organs, with particular emphasis on the role of aortic inflammation or infection in this process [11].

The proposed criteria for diagnosing primary AEF involve an atherosclerotic aneurysm or penetrating ulcer tethering to the GI tract, luminal GI contents of similar radiographic density to the aortic contents, absence of previous aortic surgery, and lack of competing explanations for GI hemorrhage or cause of death. Secondary AEF requires a history or evidence of previous aortic surgery or intervention, without the necessity of an atherosclerotic aneurysm or penetrating ulcer [12].

Diagnostic tools for aorto-enteric fistula (AEF) encompass upper gastrointestinal endoscopy (EGD) and CT scans. Despite its utility, EGD exhibits a sensitivity of less than 50 %, largely attributable to the frequent localization of fistula in the third and fourth parts of the duodenum [13]. Conversely, for hemodynamically stable patients with a known abdominal aortic aneurysm (AAA), CT angiography serves as a crucial diagnostic modality, boasting a sensitivity ranging from 50 % to 94 % and a specificity of 85 % to 100 % [[13], [14], [15], [16]]. In the living, optimal imaging assessment involves unenhanced CT, arterial phase contrast CT, and delayed imaging after 80 s [4]. Key CT findings indicative of AEF include ectopic gas within the Aorta, loss of the paraaortic fat plane, bowel wall thickening, aortic wall loss, obliteration of the fat plane between the duodenum and aorta, and contrast extravasation from the aorta into the gastrointestinal tract [4,[14], [15], [16], [17]]. Nonetheless, these findings lack sensitivity, underscoring the importance of a comprehensive approach that incorporates clinical history, particularly pertaining to aortic surgery or intervention [16]. While urgent ultrasound aids in AAA identification, it does not definitively confirm AEF, necessitating blood culture when AEF is suspected [17]. Other imaging modalities such as arteriography, magnetic resonance imaging, and colonoscopy offer limited diagnostic value [18]. In our case, considering the patient's symptoms of hematemesis, melena, generalized abdominal pain, pallor and signs of hemodynamic instability along with the history of aortic stent placement surgery raised suspicion for prompt evaluation with CT scan as the preferred diagnostic modality. The scan revealed thrombosis in the right iliac artery stent, which contains air bubbles, along with circumferential wall thickening of graft, adjacent ectopic gas, abscess formation in right psoas muscle and suspicious connection of graft to duodenum, suggestive of a secondary iliac graft-enteric fistula.

Treating secondary AEF involves prompt resuscitation, prescription of antimicrobial therapy, management of the bleeding, and, if feasible, repair of the aorta [9]. Untreated secondary AEF is nearly always devastating. Surgery appears to play a crucial role in the management of AEFs. Despite surgery, the death rate can vary from 18 % to 63 % [19]. Aside from managing blood flow and repairing blood vessels, surgery can also involve removing infected deceased tissue and restoring gastrointestinal function in cases with secondary AEFs [17].

Regular follow-ups, including upper gastrointestinal endoscopy, CT angiography of the aorta and iliac arteries, and stool occult blood tests after discharge, are essential for the early detection of complications in patients with serious conditions such as fistula and thrombosis. The positive outcomes observed during the six-month and eighteen-month follow-ups indicate the effectiveness of the interventions and the stable health status of the patient.

## 4 Conclusion

In summary, aorto-enteric fistula (AEF) can occur due to a thrombosed graft. Given the atypical nature of this fistula formation route, complications in its diagnosis may arise. Therefore, in patients with vascular manipulation and a history of gastrointestinal bleeding or anemia, AEF should be considered as a differential diagnosis. CT scan has a crucial role in achieving timely diagnosis. The evaluation and management approach for AEFs via a stent graft would be similar to the example described. It is essential to recognize that this condition can still lead to severe morbidity and necessitate a lengthy hospital stay.

## 5 Methods

This case report has been reported in line with the SCARE Criteria [20] at the end of the introductory section.

## Author contribution

All authors contributed to the study design, writing of the paper, and final approval of the case report.

## Consent

Written informed consent was obtained from the patient for publication of this case report and any accompanying images. A copy of the written consent is available for review by the Editor-in-Chief of this journal upon request. The consent was obtained in March 2024.

## Ethical approval

Ethical approval for this study (Ethical Committee No IR.GOUMS.REC.1403.155) was provided by the Ethical Committee of Golestan University of Medical Sciences, Golestan, Iran on 30 July 2024.

## Patient perspective

The patient and his companions have expressed gratitude for the early diagnosis and the treatment process, including the surgical procedure, and have commended physicians.

## Guarantor

The corresponding author is the guarantor of submission.

## Research registration number

None.

## Funding

There was no funding.

## Declaration of competing interest

None.
